# Supplementary material for: Rapid turnover of life-cycle-related genes in the brown algae
Source: Genome Biol. 2019 Feb 14;20:35. doi: 10.1186/s13059-019-1630-6 (PMC6374913; doi:10.1186/s13059-019-1630-6)
Supplement: Supplementary file 3 — Protein alignments, corresponding Gblocks html file and CDS sequences for the lineage conserved gametophyte-biased genes. (ZIP 2109 kb) [file 13059_2019_1630_MOESM3_ESM.zip › Con_GA_alignments/Ec-02_001590.aa_ali.fasta-gb.htm]

Ec-02\_001590.aa.fasta.trans.aa\_ali.fasta


## Gblocks 0.91b Results

Processed file: **Ec-02\_001590.aa.fasta.trans.aa\_ali.fasta**  
Number of sequences: **2**  
Alignment assumed to be: **Protein**  
New number of positions: **420** (selected positions are underlined in blue)

```
                         10        20        30        40        50        60
                 =========+=========+=========+=========+=========+=========+
Ec-02_001590     MAVTDNIQPQ------DSPVDEHRYAKIFEKLD-TEDGYLSGKRAVVLLRKSGVPQETLS
MSTRG.42644.1    MIATDGMRADAYLHGVSEVVDKSKYEKMFRKLNGAGDGYLDGKRAVALLSKSGLPQGTLA
                                    #############    ########################


                         70        80        90       100       110       120
                 =========+=========+=========+=========+=========+=========+
Ec-02_001590     KVWALADSDVDGRLSLKEFCTAMHLIGCFRKGLPLPEVPSRKPKKPKKRQSPGGDTKPRE
MSTRG.42644.1    KVWTLSDRDVDGRLSVKEFCEAMHLIACVRRGMPLPNLPTEAGKKKKTARSHRQSSAARA
                 ########################################################### 


                        130       140       150       160       170       180
                 =========+=========+=========+=========+=========+=========+
Ec-02_001590     GQ-GKREITFAKSPKRRSRLASLVDP-----HQDANPPGASIAKSHEPLLPLSRIRKSAT
MSTRG.42644.1    APNEARGSTSANSPKRQSRMASLTEPPHAHASQLADGPCTSTLESQNLQVSDGEVCDYLD
                      #####################      #############               


                        190       200       210       220       230       240
                 =========+=========+=========+=========+=========+=========+
Ec-02_001590     LWILTLCG-SRSPMITSQWKSRRLKELR---QHRKEAEES--------------------
MSTRG.42644.1    LRQSEQREDSVSQGVQAQPEASRLFDKKSALQTRVEAAELGKQVPGQDGTVHGNVRGTNA
                          ###############                                    


                        250       260       270       280       290       300
                 =========+=========+=========+=========+=========+=========+
Ec-02_001590     ---RDHG-------GQSRIRRPNLRQLDDKDVDAVFAEGPGAINSLRDVQSWRVLVMGLQ
MSTRG.42644.1    VSNRRQGVKNRGPVASPCKRRPKLRQLNANNVDAVYAEGTGAIDSLRDVQSWRVLVMGLE
                                    #########################################


                        310       320       330       340       350       360
                 =========+=========+=========+=========+=========+=========+
Ec-02_001590     RLSGKDMARHVPTNVRPRRRHTLPLAAKGDLARKQDYLPGRGRRTSLLGTAYEPAALFLK
MSTRG.42644.1    RLFGKDMARRVPVQARLRRRHTLPMATEGGIARKLEFLPGGGRRTSLLGTAYEPAAFFLR
                 ############################################################


                        370       380       390       400       410       420
                 =========+=========+=========+=========+=========+=========+
Ec-02_001590     QRAERRSIREADLEKDTAKAAEKWNLKPLREKSERCES---DGEQTSSR-GNLSVAGLPL
MSTRG.42644.1    QRAERRSAREIDLEKDTAKAAEKWSLQPLEDNAERDASGAIGGEDKANRRGNLSVAGLPL
                 ######################################            ##########


                        430       440       450       460       470       480
                 =========+=========+=========+=========+=========+=========+
Ec-02_001590     LDEDVDIIVHLLRQNHAFKRLDLSRCFLSEKQFVSLAQALAWNSRIEELFLQECVFTSVG
MSTRG.42644.1    LDEDIDVIVDLLRQNHAFRRLDLSRCFLSEGQFVALAQALAWNSRIEELCLQECVFTPAG
                 ############################################################


                        490       500       510       520       530       540
                 =========+=========+=========+=========+=========+=========+
Ec-02_001590     TAALADVLTVNNHIHSLDVRGCKGLDEKSIKVILGGARSKGNLLTFNGMNLADLRAEGSD
MSTRG.42644.1    AAALADVMTMNNHITSIDVRGCKGLNEDGIKVILRASRKKAKLSTFNGMKLAALRAEGSD
                 ############################################################


                        550       560       570       580       590       600
                 =========+=========+=========+=========+=========+=========+
Ec-02_001590     ELDLSG------------------------------------------------------
MSTRG.42644.1    ELDLSGSGLGYVEAVVVADCLRSRPDGLESVNFSNNSLSTEAFTTLMVALRKQSQLRSLD
                 ######                                                      


                        610       620       630       640       650       660
                 =========+=========+=========+=========+=========+=========+
Ec-02_001590     ------------------------------------------------------------
MSTRG.42644.1    LSHNLVDERTLNGITELLHRNPKLASVNLTDNRISCNEFEHCGLDLMRLLSSNNTITVFN
                                                                             


                        670       680       690       700       710       720
                 =========+=========+=========+=========+=========+=========+
Ec-02_001590     ------------------------------------------------------------
MSTRG.42644.1    VEENDVSPKMKSIIGEKLAANRAVRKDMTFVDCLHLKYPRVAPRPVLADPRRLHLADADV
                                                                             


                        730       740       750       760
                 =========+=========+=========+=========+=====
Ec-02_001590     ---------------------------------------------
MSTRG.42644.1    PFMIQEGIRAVQTTVRTEQATIATLDGGKPGSPYDLFFVQRCNNV
```

```
Parameters used
Minimum Number Of Sequences For A Conserved Position: 2
Minimum Number Of Sequences For A Flanking Position: 2
Maximum Number Of Contiguous Nonconserved Positions: 8
Minimum Length Of A Block: 10
Allowed Gap Positions: None
Use Similarity Matrices: Yes
```

```
Flank positions of the 7 selected block(s)
Flanks: [20  32]  [37  119]  [126  146]  [153  165]  [190  204]  [260  398]  [411  546]  

New number of positions in Ec-02_001590.aa.fasta.trans.aa_ali.fasta-gb:  420  (54% of the original 765 positions)
```
